# Recent Developments in Targeting RAS Downstream Effectors for RAS-Driven Cancer Therapy

**DOI:** 10.3390/molecules26247561

**Published:** 2021-12-14

**Authors:** Ozge Tatli, Gizem Dinler Doganay

**Affiliations:** 1 Department of Molecular Biology, Genetics-Biotechnology, Graduate School, Istanbul Technical University, Istanbul 34469, Turkey; tatlio@itu.edu.tr; 2 Department of Molecular Biology and Genetics, Istanbul Medeniyet University, Istanbul 34720, Turkey; 3 Department of Molecular Biology and Genetics, Istanbul Technical University, Istanbul 34469, Turkey

**Keywords:** RAS-driven cancers, RAS effectors, Raf/MEK/Erk, PI3K-mTOR

## Abstract

Aberrant activity of oncogenic rat sarcoma virus (RAS) protein promotes tumor growth and progression. RAS-driven cancers comprise more than 30% of all human cancers and are refractory to frontline treatment strategies. Since direct targeting of RAS has proven challenging, efforts have been centered on the exploration of inhibitors for RAS downstream effector kinases. Two major RAS downstream signaling pathways, including the Raf/MEK/Erk cascade and the phosphatidylinositol-3-kinase (PI3K) pathway, have become compelling targets for RAS-driven cancer therapy. However, the main drawback in the blockade of a single RAS effector is the multiple levels of crosstalk and compensatory mechanisms between these two pathways that contribute to drug resistance against monotherapies. A growing body of evidence reveals that the sequential or synergistic inhibition of multiple RAS effectors is a more convenient route for the efficacy of cancer therapy. Herein, we revisit the recent developments and discuss the most promising modalities targeting canonical RAS downstream effectors for the treatment of RAS-driven cancers.

## 1. Introduction

Rat sarcoma virus (RAS) is involved in distinct cellular processes, including cell division, proliferation, migration and cellular differentiation. RAS is tethered to the plasma membrane and acts as a nexus to relay mitogenic stimuli from receptor tyrosine kinases (RTKs), thereby activating a plethora of downstream signaling pathways. RAS proto-oncogenes encode four members of evolutionary conserved, membrane-associated small GTP-binding proteins (HRAS, NRAS, KRAS4A, KRAS4B). The activity of RAS is tightly regulated by a molecular switch between GTP-bound active and GDP-bound inactive conformation. Two classes of regulatory proteins maintain the GTPase activity of RAS proteins: guanine exchange factors (GEFs) catalyze the exchange of GDP for GTP, and GTPase activating proteins (GAPs) dictate the spatial regulation of RAS by accelerating the hydrolysis of bound GTP to GDP and inorganic phosphate [1].

RAS hyperactivation is frequently observed in a large spectrum of cancer types, including pancreatic ductal adenocarcinomas (PDACs), colorectal myelomas, lung adenocarcinomas and endometrial carcinomas. RAS-activating mutations and epidermal growth factor (EGF) hyper-signaling, which potentiates cell proliferation, are common in cancer. RAS proteins are overactive in nearly 30% of all cancers and are associated with resistance to frontline monotherapies [2]. Among RAS isoforms, KRAS mutations are most frequently found in pancreatic, colorectal and lung adenocarcinomas, while NRAS and HRAS are generally mutated in some melanomas, leukemias and thyroid cancers [3]. Mostly, RAS genes harbor oncogenic mutations which abrogate intrinsic GTPase activity of RAS protein and drive tumorigenesis. In all three RAS isoforms, G12, G13 and Q61 sites are the three mutational hotspots, which consist of 98% of all known RAS mutations. Mutations on these sites lead to the expression of constitutively active RAS proteins that induce cellular transformation and tumorigenesis [4,5]. Except for these mutations, abnormal activity of RAS can also arise from GDP-GTP deregulation, loss of GAPs or RTK-mediated activity of GEFs.

In the last three decades, tremendous efforts have been expended on targeting RAS-driven tumors. In an effort to inhibit mutant RAS function, RAS can be directly targeted by molecules disrupting its interaction with SOS1 (SOS Ras/Rac Guanine Nucleotide Exchange Factor 1) or with its effectors such as Raf and PI3K (phosphatidylinositol-3-kinase). However, RAS proteins are often termed “undruggable” due to the consecutive failures in the development of direct RAS inhibitors. Various alternative ways to battle against this perception have been implemented [6,7,8]. Thus far, only the KRAS^G12C^ mutant has been tractable in blocking the oncogenic signal [9,10,11,12]. In 2013, Ostrem et al. demonstrated that mutant RAS can be selectively targeted through a new allosteric regulatory site defined on RAS. The researchers identified a covalent compound that could bind to the mutant cysteine on KRAS^G12C^ via a disulfide bond and inhibit SOS1-catalyzed nucleotide exchange [13]. In the search for an inhibitor with a better binding and pharmacologic property, ARS-1620 has been developed as a RAS inhibitor [12] and revealed variable responses in a panel of KRAS^G12C^ mutant cell lines, which can be enhanced by concomitant inhibition of the PI3K cascade [14,15]. Further, newer molecules sotorasib (AMG-510) and adagrasib (MRTX849), which are structural derivatives of ARS-1620 with enhanced sensitivity, have been developed and entered the clinic [9]. However, given the variability of response in tumor models, direct KRAS^G12C^ inhibitors will not likely confer a benefit to a significant number of patients.

Oncogenic RAS signaling can be blocked indirectly by targeting the attachment of RAS to the inner plasma membrane or inhibiting RAS-dependent metabolic processes such as autophagy and macropinocytosis [16]. A variety of post-translational modifications of cytosolic precursor RAS protein regulate its membrane association and activation. These modifications include prenylation, post-prenylation, palmitoylation, ubiquitination, phosphorylation, SUMOylation, acetylation, nitrosylation, etc. Among them, the processes of prenylation and post-prenylation that mediate membrane localization are major potential therapeutic targets [17]. In particular, molecules targeting farnesylation of RAS via CAAX mimetic polypeptides or farnesyl pyrophosphate analogs have been clinically evaluated for the treatment of RAS-mutant advanced solid cancer [18,19]. However, the effectiveness of these inhibitors for RAS-driven solid cancer has been less than favorable in single-agent settings. Similarly, a number of studies show that the inhibition of one particular modification remains insufficient to prevent tumorigenesis [20,21].

Targeting RAS effector signaling cascades or synthetic lethal interactors of mutant RAS are other indirect strategies being pursued to conquer RAS-mutant cancers [22]. The former is one of the most dynamic arms in the development of RAS-inhibitory molecules. However, RAS downstream effector signaling is far from consisting of linear unidirectional cascades. Conversely, it can adapt and rewire in response to the blockage of specific nodes. Therefore, cancer cells develop dependencies upon inhibition of only RAS or a single RAS effector, which contribute to acquired drug resistance in single-agent therapies. For the efficacy of cancer therapy, synergistic inhibition of specific targets is increasingly adopted to block compensatory mechanisms between these two pathways. Despite the advances in targeted therapies in the treatment of cancer, acquisition of drug resistance still remains a major drawback. In this review, we revisit the recent strategies and discuss the most promising approaches targeting canonical RAS downstream effectors for the treatment of RAS-induced cancers.

## 2. Overview of the Downstream Signaling Pathways of RAS

**Figure 1:**
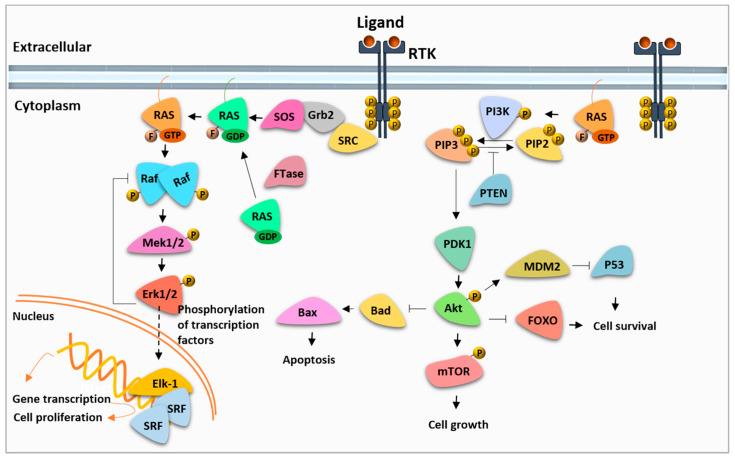
Ras/Raf/MEK/Erk and PI3K/mTOR signaling pathways.

RAS engages with two major downstream effector signaling pathways, including Raf/MEK/Erk (MAPK) and phosphatidylinositol-3-kinase (PI3K) cascades. These pathways are associated with specific cancer-related phenotypes, including transcriptional reprogramming, promoted cell survival and proliferation, suppressed apoptosis and enhanced invasiveness of the cells [23]. Mitogenic signals from upstream growth factor receptors are transmitted into cells by RAS proteins. RAS proteins attach to the plasma membrane with a process facilitated by several post-translational modifications. A farnesyl isoprenoid moiety is added to RAS by farnesyltransferase (FTs) to increase its hydrophobicity and enable its association with the plasma membrane [24]. Membrane-anchored RAS is activated by GTP loading and serves as an activator for effector kinases by recruiting them to the cell membrane, where further phosphorylation events enable their subsequent activation [25]. GTP-loaded RAS proteins can activate both PI3K/Akt/mTOR and Raf/MEK/Erk signaling cascades, which are compelling therapeutic targets as major mediators of RAS-induced oncogenic signal transduction (Figure 1).

The RAS-Erk signaling network, which is the central regulator for cell cycle progression and proliferation, is a prominent mediator of RAS-dependent cancer growth. RAS stimulates cell proliferation by increasing the concentration of Raf kinases to the plasma membrane, where effector kinases are activated [26]. Here, active RAS drives Raf dimerization by promoting conformational changes, which further trigger dephosphorylation of the inhibitory sites and phosphorylation of activatory sites [27]. Active Raf in turn activates MEK through phosphorylation, which results in the final activation of Erk. Following activation, Erk translocates to the nucleus, where it activates several transcription factors and cell cycle regulatory proteins. The cascade is modulated by a large number of factors [28,29]. Particularly, Erk does not only activate its downstream substrates, but also inhibits the upstream kinases in the MAPK pathway through phosphorylation. Thus, it can modulate the pathway both negatively and positively through feedback inhibition. Among the three MAP kinases, Raf kinases are the most critical effectors in KRAS-driven cancers. B-Raf is one of the most mutated Raf isoforms in cancerous cells. The well-known driver mutation in B-Raf occurs at the position of V600. In this mutant form, B-Raf is constitutively active, independent of upstream cues [30,31].

The other well-described downstream pathway of RAS is the PI3K-mTOR signaling cascade, which controls a number of cellular events including cell cycle progression, protein synthesis, metabolism and survival [32]. PI3K signaling can be activated by RAS, receptor tyrosine kinases (RTKs) or G protein-coupled receptors (GPCRs). Phosphatidylinositol kinases (PI3K) are intracellular lipid kinases that phosphorylate phosphoinositides and generate biologically active phosphatidylinositol 3,4,5-triphosphate (PIP3). PIP3 recruits PDK1 and Akt to the plasma membrane, where they are subsequently activated. Active Akt propagates signals to several substrates, including mammalian target of rapamycin (mTOR), forkhead box O (FOXO) or nuclear factor (NF)-κB, which induce the protein synthesis, cell growth and glucose and lipid metabolism. The pathway is negatively regulated by lipid phosphatase PTEN, which dephosphorylates PIP3 and thereby reduces the level of phosphorylated Akt. Once Akt is deactivated by protein phosphatases PHLPP1/2 and PP2A, the signaling events are terminated [33,34]. Both RAS effector pathways are modulated by a series of protein kinases, phosphatases and multiple exchange proteins, and they influence each other in both negative and positive ways. Therefore, in the case of chemical inhibition of one pathway, the effect of the inhibitor is compensated in the cell by the activation of the other pathway [35,36,37,38]. Single-agent therapies generally fail to produce durable responses due to the incomplete apoptosis and development of resistance to the targeted agent by compensatory mechanisms [39,40]. The crosstalk between these two pathways forms a basis for combinatorial therapies in cancer progression [36]. Therefore, delineating the intricate underlying molecular mechanisms of the extensive crosstalk between these signaling cascades is of great importance for effective targeted therapeutic strategies. 

### 2.1. Raf Inhibitors

So far, three B-Raf kinase inhibitors, including vemurafenib [41,42], dabrafenib [43,44] and encorafenib [45], have been clinically approved by the US Food and Drug Administration (FDA) for selective inhibition of kinase activity in B-Raf^V600^ mutant-driven melanoma. These inhibitors hinder catalytically active B-Raf^V600^ mutant monomers with specificity for the ATP-binding site, but not non-V600 mutants [46,47,48]. However, monotherapy with these inhibitors has been shown to promote paradoxical activation of Erk by inducing wild-type Raf dimerization in non-tumorous cells and also RAS-induced tumors which signal through Raf dimers [49]. When Raf proteins are dimerized, the inhibitor-bound Raf protomer allosterically transactivates the other Raf component of the dimer, thereby resulting in the final activation of Erk.

In the presence of oncogenic RAS, B-Raf inhibition has been shown to lead to the formation of RAS-dependent B-Raf/C-Raf heterodimers and drive tumorigenesis through C-Raf activation [50]. A significant number of patients on B-Raf-inhibitor therapy developed secondary malignancies, most commonly cutaneous squamous-cell carcinomas (cuSCC), due to the intrinsic or acquired drug resistance at tolerable doses [51]. Thus, B-Raf inhibitors alone have not been used effectively for the therapy of RAS-addicted cancers. Instead, combining B-Raf inhibitors with MEK inhibitors has been shown to be useful not only to provide a better survival outcome, but also to reduce the incidence of cuSCC [52,53].

In an effort to suppress B-Raf activity without stimulating cancer growth, second-generation Raf inhibitors including type II pan-Raf inhibitors and paradox-breakers were developed to hinder dimerization-driven transactivation. These inhibitors possess minimal paradoxical activation compared to approved B-Raf^V600^ inhibitors through distinct mechanisms [54]. Of note, the major concern for the clinical use of pan-Raf inhibitors is their lack of selectivity for mutant B-Raf, which might cause toxicity resulting from the blockade of MAPK signaling in normal tissue. Pan-Raf inhibitors bind to both protomers in Raf dimers and lock them in an α-helix-in, DFG-out conformation [55,56]. Examples of pan-Raf inhibitors include sorafenib, belvarafenib, AZ-628, CCT196969, CCT241161, LY3009120, LXH254 and TAK-580. Apart from these, CCT3833 works as a dual pan-Raf inhibitor by inhibiting both Raf and upstream kinases of RAS, thereby preventing RAS activation by the relief of negative feedback loops [57,58]. Several pan-Raf inhibitors showed promising results in preclinical models of cancers bearing RAS mutations, including melanoma [54], acute myeloid leukemia (AML) [59], colorectal cancer [60], multiple myeloma [61,62], pancreatic cancer [63] and thyroid cancer [64], and are currently under clinical evaluation. Dual pan-Raf inhibitors, CCT196969 and CCT241161, that also target SRC (SRC Proto-Oncogene, Non-Receptor Tyrosine Kinase) as one of major MAPKs regulatory protein tyrosine kinases, inhibited the growth of NRAS-mutant melanoma cells and achieved tumor regression in patient-derived xenografts that are resistant to B-Raf inhibitors. These findings suggested that they could provide clinical benefit in melanoma patients with NRAS mutations as a first-line therapy and in relapsed patients as a second-line therapy [65]. However, it is important to take into account that benefits in preclinical studies have not always translated into clinical success. For example, LY3009120 has been shown to be effective in numerous in vitro studies and preclinical models [60,63,66], but exhibited unexpectedly limited pharmacodynamic effects at its maximum tolerated dose in patients with B-Raf or KRAS mutations (NCT02014116) [67]. Unlike LY3009120, CCT3833 significantly prolonged the survival of a patient with KRAS^G12V^ mutant spindle cell sarcoma which was refractory to multi-kinase inhibitor treatment in a phase I clinical trial (NCT02437227) [68]. Similarly, another pan-Raf inhibitor, lifirafenib (BGB-283), elicited an acceptable safety profile and clinical efficacy in patients with KRAS-mutated NSCLC and endometrial cancer (NCT02610361) [69]. These results have encouraged a phase I/II trial of lifirafenib in combination with the MEK inhibitor mirdametinib (PD-0325901) in patients with B-Raf- and RAS-mutant tumors (NCT03905148). In addition, LXH254, a novel type II pan-Raf inhibitor developed by Novartis, has been shown to be potent, selective, efficient and well-tolerated in RAS-mutant xenograft models [70]. Moreover, LXH254 inhibited long-term cell viability in NRAS-mutant NSCLC cells when combined with volasertib, a polo-like kinase inhibitor [71]. LXH254 is being further investigated in combination with a MEK inhibitor (trametinib), an Erk inhibitor (LTT462) or a CDK inhibitor (ribociclib) in patients with NRAS mutant melanoma (NCT02974725, NCT04417621). Another pan-Raf inhibitor, Belvarafenib, demonstrated anti-tumor activity as a single agent and has been found to be well-tolerated in patients with Raf or RAS mutations (NCT03118817) [72]. Clinical studies utilizing pan-Raf inhibitors in combination with other therapeutic agents to improve the response rate within patients with RAS mutations are ongoing (NCT02607813, NCT04835805, NCT04059224, NCT03284502).

The efficacy of pan-Raf inhibitors is promising, but nevertheless not striking for anti-RAS cancer therapy, which requires excellent drug combination for a robust response from dozens of alternatives. In this regard, Rukhlenko et al. built a system biology-based dynamic model to analyze the synergy of Raf inhibitor combinations in an oncogenic RAS and/or B-Raf^V600E^ background, taking into account the thermodynamics and kinetics of inhibitor–protein interactions, post-translational modifications and structural determinants [73]. They found that two distinct conformation-specific kinase inhibitors targeting the same kinase but in different conformations could block the paradoxical activation. The synergy of structurally different Raf inhibitors was validated in mutant NRAS, HRAS and BRaf^V600E^ cells [73]. The data demonstrated that the combination of an α-C helix in/DFG loop-out inhibitor (e.g., sorafenib, LY3009120) and an α-C helix-out/DFG loop-in inhibitor (e.g., vemurafenib or dabrafenib) has effectively targeted cells with both B-Raf^V600E^ and RAS mutations [73], thus providing a potential to target tumors with such genetic backgrounds.

Another class of B-Raf inhibitors, paradox breakers, have been developed utilizing the structure of vemurafenib as a skeleton, with various chemical modifications (Table 1 and Table S1). Candidate compounds were screened for hindering B-Raf^V600E^ and evaluating pathway reactivation in RAS-mutant cell lines. These inhibitors dock on Raf proteins to impair Raf dimerization [74,75,76] and prevent the formation of B-Raf/C-Raf heterodimers observed in RAS-mutant tumors treated with first-generation Raf inhibitors [56]. The paradox breakers, PLX7904 and PLX8394, have been reported to possess a more durable efficacy in cuSCC mouse cells with a HRAS^Q61L^ mutation, compared to vemurafenib [76]. In a later study, it has been shown that PLX8394 is able to selectively disrupt B-Raf homodimers and B-Raf/C-Raf heterodimers, but C-Raf homodimers are unresponsive to PLX8394 due to its inability to selectively bind C-Raf [77]. Given that C-Raf fusions can activate both the MAPK and PI3K/mTOR signaling cascades, the efficacy of these drugs most likely will not be sufficient in a single-agent setting for the treatment of tumors driven by RAS mutations due to the possible induction of C-Raf fusion formation.

Transmission of signals from B-Raf to MEK requires the dimerization of B-Raf protomers in both normal cells and non-B-Raf^V600^ tumor cells [78]. To circumvent the transactivation of Raf dimers elicited by current Raf inhibitors, the dimer interface can be targeted by allosteric B-Raf inhibitors, thereby eliminating overactive MAPK signaling induced by oncogenic B-Raf or RAS. The dimer interface of B-Raf protomers exists in the kinase domain of B-Raf at the C-terminal end of the αC helix. R509 residue plays the central role to provide dimer integrity. Mutations in R509/L515/M517 residues have been shown to completely block the activity of wild-type B-Raf [79]. To target B-Raf dimers, Beneker et al. have developed type IV inhibitors and designed a dimeric disrupter peptide to allosterically inhibit Raf kinase activity. This strategy enabled the blockade of paradoxical activation induced by vemurafenib [80]. Similarly, Gunderwala et al. have shown that in silico designed B-Raf dimer-breaking peptides induce proteasome-mediated degradation of B-Raf and inhibit kinase activity in KRAS mutant tumor cells. Additionally, a combination of these peptides with ATP-competitive inhibitors provided a by-pass of the cellular compensatory mechanisms caused by ATP-competitive Raf inhibitors [81]. In a subsequent study, Raf dimer breaker has been shown to be active against oncogenic B-Raf^D594G^:C-Raf dimers [82], thus providing further evidence that allosteric type IV inhibitors targeting the Raf dimer interface have potential to be developed as an anticancer drug.

Among Raf isoforms, B-Raf has taken the center stage in targeted cancer therapy due to its high incidence of mutations in various types of cancer. Remarkably, another member of the Raf family, C-Raf (also known as Raf1), has been shown to have an essential role to relay upstream signals to MEK and Erk in K-RAS^G12V^-addicted non-small-cell lung cancer (NSCLC) in mice. While the ablation of C-Raf from these cells inhibited tumor development, B-Raf did not show the same effect, indicating that the loss of C-Raf cannot be compensated by other Raf isoforms in K-RAS^G12V^-addicted NSCLCs [83]. In line with this finding, systemic ablation of C-Raf and EGFR has been shown to induce tumor regression in pancreatic ductal adenocarcinoma models with KRAS/Trp53 mutations [84]. The loss of C-Raf expression did not affect MAPK signaling and possessed limited toxicity in mice [85]. Disappointingly, Morgan et al. have recently reported that selective inhibition of C-Raf elicited transactivation in engineered HCT116 KRAS^G13D^-mutant cells [86]. Therefore, as McCornick pointed out, targeting the stability of C-Raf and triggering its degradation might be an alternative strategy to avoid promoted paradoxical activation [87]. On the other side, Nolan et al. have recently reviewed the kinase-independent functions of C-Raf and explored targeting its effectors, in particular proapoptotic proteins ASK1 and MST2, and suggested that disruption of these protein–protein interactions or design of kinase activators in the context of ASK1 or MST2 activation might be a new avenue for anti-RAS cancer therapy [88].

### 2.2. MEK Inhibitors

Since attempts to target Raf kinases have suffered from acquired drug resistance that limits the effectiveness of inhibitors, the attention turned to the inhibition of MEK kinase. Many kinase inhibitors have been developed to compete for ATP by directly binding to this conserved site, which limits the selectivity of the molecule. Therefore, a number of allosteric MEK inhibitors have been developed for enhanced selectivity. These MEK inhibitors bind to a unique site close to the ATP-binding pocket of MEK, not competitively to the ATP-binding site. Thus, allosteric MEK inhibitors only bind to MEK selectively and inhibit the function of the kinase by inducing a conformational change that locks the enzyme in a catalytically inactive state [89]. High selectivity yields lower toxicity, and improved physicochemical properties. Three allosteric MEK inhibitors, trametinib, cobimetinib and binimetinib, are approved for treatment of patients with B-RafV^600E/K^ melanoma. However, tumor responses to MEK inhibitors have been generally transient due to the rapid emergence of resistance in RAS-addicted cancers. Furthermore, in a subset of cancers in which MEK inhibitors have shown a notable clinical activity, patients have suffered from off-target effects, including dermatological and gastrointestinal toxicities [90]. Inhibition of MEK has been reported to lead to some resistance mechanisms, including the feedback activation of the PI3K pathway [91] and reactivation of the MAPK pathway [92,93]. The former has been shown to be stronger in RAS-mutant cancers. The difference in the sensitivity to MEK inhibitors between RAS- and B-Raf-mutant cancers might arise from the level of dependency on Erk-mediated mTORC1 activation in tumors bearing RAS mutations [94]. For example, trametinib has failed to confer a superior clinical benefit against KRAS-mutant NSCLC over docetaxel alone (NCT01362296) [95]. Similarly, selumetinib, which is an oral, potent MEK inhibitor, has not shown a significant efficacy in the treatment of NSCLC with KRAS mutations (NCT01229150, NCT01933932) [96,97]. In NRAS-mutant melanoma patients, Binimetinib treatment also did not provide any measurable benefit as monotherapy (NCT01763164) [98]. However, KRAS^G12C^ tumors have been shown as more sensitive to selumetinib compared to KRAS^G12D^ in lung cancer mouse models. This finding underscores the heterogeneity of tumors harboring different KRAS mutations [99].

Due to the limited efficacy of MEK inhibitors in mono-agent settings, efforts have focused on the development of combinatorial strategies. MEK inhibitors have been used in combination with other treatment modalities in RAS-mutant cancers, such as conventional chemotherapeutic agents [100], systemic immunotherapies [101,102] and Raf inhibitors [103,104] to sustain a prolonged clinical benefit. Combined treatment of trametinib and immunomodulatory antibodies has shown preclinical efficacy in KRAS/p53-mutant lung cancer, suggesting a potential therapeutic approach using MEK inhibitors and immunotherapies [105]. The inactivation of zeste homolog 2 enhancer (EZH2), which is a histone methyltransferase regulating the expression of a variety of genes, has been linked to RAS signaling [106]. Owing to this, Lorenz Berg et al. have shown that RAS-mutant myeloid leukemia cells were sensitized to MEK inhibitors upon EZH2 inactivation. Thus, co-inhibiting EZH2 and MEK might provide a novel therapeutic route for RAS-driven cancers [107]. BI-3406, a novel SOS1-KRAS interaction inhibitor designed to target the catalytic domain of SOS1, has been combined with trametinib and sensitized KRAS-driven cancers to MEK inhibition in mouse models. MEK-induced drug resistance was attenuated in both G12 and G13 variants, which account for 80% of all KRAS-dependent cancers [108]. Similarly, co-targeting MEK and SHP2, which is required for RAS activation, has provided clinical utility and impaired cancer cell growth in both in vitro and in vivo settings of KRAS-mutant pancreatic cancer and NSCLC [109] and gastroesophageal cancer [110].

A unique Raf/MEK inhibitor, VS-6766/CH5126766, has shown activity in xenograft models of RAS-mutated cancers [111,112]. In parallel, combined treatment with VS-6766/CH5126766 and AXL inhibitor (bemcentinib) has provided a stronger blockage of tumor growth in KRAS-mutant ovarian cancer cells with overexpressed AXL [113]. This allosteric inhibitor prevents the release of MEK from Raf, and thus blocks the subsequent phosphorylation of both MEK and Erk [114]. VS-6766/CH5126766 has recently gone to clinical trials in patients with KRAS-addicted lung and gynecological cancers. Initial results from the phase 1 dose-escalation and basket dose-expansion study have been encouraging due to the tolerability and clinical activity of VS-6766/CH5126766 in patients (NCT02407509) [115]. From the same trial, it has been recently reported that VS-6766 is effective in halting cancers with non-G12C-KRAS mutations when administered in an intermittent schedule [116]. The intermittent regimen has been previously shown to improve the pharmacokinetic/pharmacodynamic profile of VS-6766 compared to continuous dosing schedules [117]. Since the therapeutic index of single-agent inhibitors is narrow in particularly RAS-driven cancers, these findings are among the most promising, reported as single-agent therapy in cancers with RAS mutations. On the basis of these findings, multiple strategies using VS-6766/CH5126766 alone (NCT03681483) or with other agents such as defactinib (NCT03875820, NCT04625270, NCT04620330) are currently under clinical investigation in cancers with KRAS mutations. Furthermore, a great majority of ongoing clinical trials are testing MEK inhibitors with or without other agents in the treatment of RAS-mutant cancers (Table 2). Among them, the CDK4/6 inhibitor palbociclib is of particular interest in RAS-mutant NSCLC due to its sensitizing role to MEK inhibitors [118]. Given that cyclin-dependent kinases (CDKs) are frequently altered in most human cancers, patients may benefit from the therapeutic use of CDK inhibitors in combination with MEK inhibitors [119].

### 2.3. Erk Inhibitors

The final kinase component of the three-layered MAPK cascade, Erk, is another target to evade compensatory resistance mechanisms. Several ATP-competitive Erk1/2 inhibitors, including ASN007 [124], LY3214996 [125], GDC-0994 [126] and MK-8353 [127], have been discovered, and some of them demonstrated a significant anti-tumor activity in tumors bearing RAS mutations. However, the therapeutic index of Erk inhibitors remains limited in monotherapies due to their inhibitory role in both malignant and normal tissues. Instead, they have been generally explored in combined modality settings to potentiate their effectiveness in cancers with RAS mutations.

Ravoxertinib (GDC-0994) is an orally available small molecule targeting ERK1/2 kinase [128] with promising preclinical efficacy findings from both in vitro and in vivo experiments [129,130]. These findings have led to the progression of GDC-0994 through human clinical trials. Disappointingly, in a phase I clinical trial, GDC-0994 monotherapy has not provided a significant clinical benefit at tolerable doses in patients with KRAS mutations [131]. In another clinical study, the effect of the compound has been evaluated in combination with cobimetinib, however the phase I study was terminated early due to the intolerability of the combination with dose-limiting toxicities of myocardial infarction and rash (NCT02457793) [132]. LY3214996, which is a novel and highly selective small-molecule inhibitor of Erk1/2, has been shown to exhibit robust anti-tumor activity in xenograft models of RAS-mutant lung cancer when administered in an intermittent regimen [125]. Consistently, LY3214996 exhibited a well-tolerable and synergistic activity profile in xenograft models of KRAS-mutant NSCLC and colorectal cancer in combined modality settings [133,134]. On the basis of its preclinical efficacy and the rationale provided by the combinatorial studies, LY3214996 was advanced into human clinical trials in cancers with RAS mutations (NCT02857270). Another Erk1/2 inhibitor, ASN007, which is a potent and selective biomolecule with a long target residence time, showed promising anti-tumor activity in vitro. It impeded tumor growth in xenograft models with B-Raf and RAS mutations [135] and also synergized with PI3K inhibitors both in vitro and in vivo [124]. Given that ASN007, alone or in combination with other agents, is expected to provide a therapeutic option for RAS-mutant cancers, it has gone to human clinical trials (NCT03415126). However, data about clinical activity are not yet available. Ulixertinib (BVD-523) is another Erk1/2 inhibitor that has been shown to suppress tumor growth and induce tumor regression in B-Raf and RAS-mutant xenograft models [136,137,138]. In a phase I dose-escalation and expansion study, ulixertinib exhibited a tolerable safety profile with promising pharmacodynamic effects in NRAS- and B-Raf-mutant solid tumors (NCT01781429). Clinical studies are ongoing to examine its role in tumors harboring activating MAPK mutations as a single agent or in combination with other agents (NCT03698994, NCT04145297). Recently, the combination of AZD0364, a selective Erk1/2 inhibitor, and selumetinib has been shown to alleviate tumor progression in multiple xenograft models of KRAS-mutant NSCLC [139]. Similarly, Catalano et al. showed that dual inhibition of MEK and Erk represented anti-tumor efficacy and blocked the emergence of drug resistance in an HRAS^G12C^-driven autochthonous sarcoma model. However, this combination could not be successful to revert previously developed resistance, which offers the use of dual MEK and Erk treatment as a first-line therapy [140]. In addition to these, the authors noted that some cell lines resistant to combined MEK and Erk inhibition showed dependency on MAP4K4 activity, which serves MAP4K4 as a potential new therapeutic target [140]. Despite these successful examples of dual MEK and Erk inhibition in some RAS-mutant models, the potential of these combinations for clinical implementation awaits further investigation.

As another example of intra-pathway dual inhibition of the MAPK pathway, concurrent application of pan-Raf inhibitors and Erk inhibitors proved potent at low doses in cell line, organoid and rat models of PDAC with KRAS mutations [141]. Altogether, these data suggest that Erk inhibitor-anchored treatment strategies can be adopted as a therapeutic option in RAS-mutant settings. Clinical studies utilizing MK-8353 (NCT02972034), AZD0364 (NCT04305249) and LTT462 (NCT02974725) as monotherapy or in combination with other therapeutic agents are also ongoing to describe the response rate within patients with RAS mutations.

The functional activity of Erk requires its translocation to the nucleus. Cytosolic active Erk is phosphorylated by MEK on its specific regulatory residues and undergoes a conformational change that releases Erk from its anchoring proteins. Then, exposed Ser residue located within the nuclear translocation sequence (NTS) is phosphorylated, triggering the interaction of Erk and the beta-like importin, Imp7, thereby escorting Erk to the nucleus, where Erk activates a large number of targets [142,143]. In an effort to block Erk/Imp7 interaction, an NTS-derived phosphomimetic peptide (EPE) has been developed and shown to inhibit proliferation of RAS-mutant cancer cells without affecting immortalized cells [142]. In a later study, Arafeh et al. showed that concomitant inhibition of MEK and the nuclear translocation of Erk synergistically reduced the viability of some NRAS mutant melanomas [144]. These findings point to the therapeutic potential of targeting nuclear translocation of Erk1/2 for cancers with RAS-mutations.

Erk5 is a more recent member of the MAPK family, which is most similar to the Erk1/2 subfamily but reveals responses through distinct mechanisms [145]. A number of studies have reported the role of Erk5 in the lack of efficacy of MAPK inhibitors in melanomas harboring NRAS and B-Raf mutations [146,147,148]. Adam et al. have shown that inhibition of Erk5 effectively sensitized NRAS-mutant melanoma cells to MAPK inhibition. Dual targeting of MEK and Erk5 using Trametinib plus XMD8-92 inhibited the growth of NRAS-mutant melanoma cells and repressed tumor progression in NRAS-mutated melanoma xenografts [148]. Concomitant inhibition of MEK1/2 and Erk5 could offer enhanced clinical efficacy for anti-cancer therapies in patients with RAS mutations.

## 3. PI3K-Akt Signaling Inhibitors and Vertical Strategies

Aberrations in the PI3K-Akt signaling pathway have been implicated with tumorigenesis and resistance to anti-cancer therapies. Dysregulation of the PI3K signaling can occur through different mechanisms with a subset of mutations in the axis. These alterations include amplification of RTKs (EGFR and HER2), mutations in PI3K subunits (p110a, and p85a, encoded by PIK3CA and PIK3R1, respectively), loss/mutation of the phosphatase tensin homolog (PTEN), Akt overexpression or RAS hyperactivation. Singly targeting RAS-induced overactivation of PI3K-Akt signaling remains challenging due to the intrinsic resistance caused by beta catenin [149] and p90 ribosomal S6 kinase (RSK) activation [150,151], the latter as a family of Erk substrate members. Thus, inhibitors of the components of PI3K-Akt signaling have been generally combined with different agents to exert a potent anti-cancer effect.

In the PI3K-Akt signaling, PI3K is the first and direct downstream kinase effector of RAS as a potential drug target. Two major classes of PI3K inhibitors, including isoform-selective inhibitors and pan-PI3K inhibitors, have been developed to antagonize PI3K-Akt signaling. In 2019, the PIK3CA inhibitor BYL719 (Alpelisib, Novartis) was approved by the FDA for the treatment of estrogen receptor-positive (ER+) breast cancer patients with PIK3CA mutations [152]. Studies evaluating the therapeutic efficacy of BYL219 in cancers bearing RAS mutations are under ongoing investigations [153,154]. The latter type of PI3K inhibitors, pan-PI3K inhibitors, include buparlisib (BKM120), PX-866, copanlisib (BAY80), LY294002, pilaralisib (XL147), pictilisib (GDC-0941) and taselisib (GDC-0032). In a recent study, GDC-0941 and siRNA therapeutics targeting KRAS (siKRAS) have been combined to test their synergistic anti-tumor effect in ovarian cancer cell lines and in an allograft ovarian cancer model harboring KRAS mutations. Here, the simultaneous use of GDC-0941 and siKRAS co-inhibited PI3K and RAS and led to the induction of apoptosis in the animal model [155]. There have been multiple examples of clinical trials for the use of siRNAs in targeted therapies [156], yet their potential for clinical utility remains to be investigated in RAS-mutant cancers.

The second kinase member in the PI3K-mTOR axis, Akt, consists of three functionally distinct isoforms, including Akt-1, Akt-2 and Akt-3. It has been shown that RAS-induced oncogenic signals are mainly relayed through the Akt-1 isoform in KRAS-mutant lung tumors [157]. In contrast, Akt-1 inhibition induced migration and invasion in KRAS or EGFR mutant NSCLC cells, but not in KRAS/EGFR wild-type cells [158]. Considering that differential genetic background contributes to the controversial roles of Akt in cancer, the selection of patient groups for clinical trials of Akt inhibitors is of great importance to avoid an Akt-inhibition-mediated metastatic effect. Recently, inhibition of Akt-1 has been shown to induce metastasis through EGFR-mediated β-catenin nuclear accumulation in breast cancer cells, suggesting that the concomitant inhibition of Akt-1 and EGFR might be effective to limit the metastatic potential of Akt-1 inhibition [159]. It has been shown that one of the compensatory cellular mechanisms of KRAS-mutant colorectal cancer cells is the increased phosphorylation of RTKs upon Akt inhibition [160]. In this study, the combinatorial use of an allosteric Akt inhibitor (MK-2206) and different RTK inhibitors (Lapatinib, OSI-906, jnj38877605) reduced the growth of cancer cells harboring KRAS mutations. Similarly, on the basis of the finding that FGFR signaling is overactivated in advanced prostate cancer, the combined use of AZD4547 (FGFR receptor kinase inhibitor) and AZD5363 (Akt inhibitor) has led to a reduction in the proliferation of prostate cancer cells both in vitro and in vivo [161]. Given these encouraging preclinical findings, combinations of Akt and RTK inhibitors await testing in clinical trials of RAS-mutant cancer therapy.

Inhibitors targeting mTOR, one of the downstream effectors of RAS, have been clinically tested, however the majority of single-agent therapies targeting mTOR did not result in clear clinical benefits. Inhibition of mTOR has been shown to stimulate feedback activation mechanisms including MEK/Erk or Akt signaling, demanding for combinatorial treatment regimens [162,163]. A phase 1 trial is currently ongoing based on the combination of a dual Raf/MEK inhibitor and evorilumus (mTOR inhibitor) (NCT02407509) in patients with RAS/Raf-mutant solid tumors. Another strategy might be to concomitantly target mTOR with other cancer-specific dependencies. For example, the abnormal activity of histone deacetylases (HDACs), which are critical regulators of gene expression, has been associated with key oncogenic events [164]. In this regard, Malone et al. analyzed the effect of dual inhibition of HDAC and mTOR in KRAS-mutant NSCLC and showed that this combination triggered catastrophic oxidative stress and tumor regression in RAS-driven tumors [165]. In several cancer types, WEE1, which is the gatekeeper of the G2 arrest, is expressed at high levels, and its inhibition sensitizes cancer cells to DNA-damaging agents by compromising the G2-M checkpoint [166]. Owing to this, Hai et al. have shown that the combined inhibition of WEE1 and mTOR synergistically induced cytotoxicity in KRAS-mutated NSCLC cells and delayed tumor growth in xenograft models without any drug-related toxicity [167]. Together, these findings represent some tractable vulnerabilities of RAS-driven cancers and are expected to bring further benefit in the context of drug combinations. More promisingly, two independent studies have shown that the efficacy of the KRAS^G12C^ inhibitor ARS-1620 was increased when combined with mTOR and an IGF1R inhibitor [15] and PI3K inhibitor [14] in lung cancer cells and in vivo models. These results showed that inhibiting the PI3K-mTOR pathway would potentiate the effectiveness of KRAS^G12C^ covalent inhibitors and provide a therapeutic opportunity for patients with KRAS^G12C^ mutations in combined settings.

## 4. Horizontal Strategies Targeting Both RAS Effector Pathways

**Figure 2:**
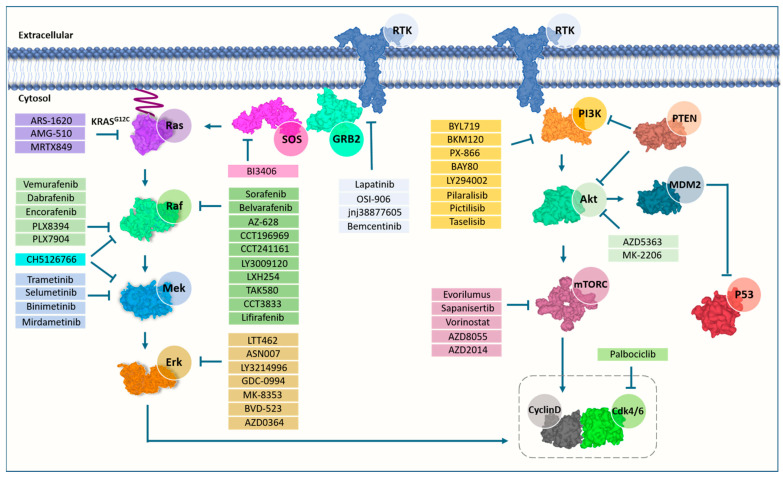
Inhibitors targeting rat sarcoma virus (RAS) effector pathways.

Owing to the adaptive resistance to selective targeting of MAPK signaling in RAS-mutant cancers, the PI3K-mTOR pathway has attracted growing interest by virtue of its complementarity to the MAPK pathway. Not only do these pathways share common inputs but also they can both be activated by oncogenic RAS and appear to provide some compensatory signaling when one or the other is inhibited (Figure 2). The therapeutic index of inhibitors targeting a single component is limited by this crosstalk, which provides numerous possibilities for overcoming the effects of inhibition. Horizontally targeting multiple effector arms might be effective to increase the therapeutic potential of inhibitors [36]. Since preclinical studies demonstrated that dual inhibition of MEK and Akt can abolish RAS signaling [168,169], selumetinib and MK-2206 have been combined to evaluate drug toxicities and anti-tumor activity in patients with KRAS mutations. Patients revealed significant treatment benefits at pulsatile dosing, which mitigate toxicities [170]. In line with this finding, it has been previously reported that different dosing regimens might change anti-tumor activity and off-target toxicities, particularly in combined modality settings which suffer from adverse effects [171]. Disappointingly, in a phase I trial, combining MEK and PI3K inhibitors have failed to determine the maximally tolerated dose due to a high incidence of adverse effects in patients with KRAS, NRAS, B-Raf or PIK3CA mutations, whereas they have shown synergistic pharmacodynamic tumor activity (NCT01392521) [172]. On the other side, blockade of RAS downstream effectors in conjunction with other cellular targets involved in RAS-driven cancers may also reveal a powerful punch. For example, Ischenko et al. showed that targeting histone deacetylases (HDACs) in combination with MEK and PI3K induced apoptosis and prevented the development of lung metastases in vivo [173]. Another drug trio, targeting MEK (PD-901), CDK4/6 (palbociclib) and mTORC1/2 (AZD2014), has reduced colony formation and S6 phosphorylation in MEKi/CDK4/6i-resistant melanoma, and significantly induced apoptosis in NRAS-mutant melanoma, but it is of note that the treatment success might be hindered by increased toxicities [174].

Tumor radio-resistance remains a major obstacle for the treatment of many types of cancer. Both MAPK and PI3K-mTOR pathways have been implicated with radio-resistance of RAS-dependent cancer cells [175,176]. Clinical data show that most cancer patients bearing KRAS mutations have a higher frequency of metastasis and recurrence of disease following radiotherapy [177]. Activation of the PI3K-Akt pathway has been shown to enable the repair of double-stranded breaks formed by radiotherapy through the induction of DNA-PKcs in cancer cells [178,179,180]. Akt-1 directly binds to DNA-PKcs and promotes its activity to initiate the NHEJ repair pathway, thereby resulting in the resistance to radiotherapy. Further, it has been shown that PI3K inhibition stimulates MEK/Erk-dependent reactivation of Akt, thus targeting PI3K alone most likely will not be sufficient to sensitize KRAS-mutant NSCLC cells to irradiation. Correspondingly, dual targeting of PI3K and MEK efficiently improved radio-sensitization through the blockade of Akt reactivation and impairment of DSBs repair [181]. In a phase Ib dose-escalation study, a highly selective pan-PI3K inhibitor, buparlisib, in combination with the MEK1/2 inhibitor trametinib, represented promising anti-tumor activity in patients with KRAS-mutant ovarian cancer; however, it should be noted that the combination required dose modifications and interruptions due to the toxicity [182].

Recently, research has focused intensively on Erk inhibitor-based drug combinations, which seem more attractive due to their ability to mitigate feedback relief. Dual inhibition of Erk and PI3K/mTOR with LY3214996 and LY3023414 inhibitors respectively, has been tolerable and resulted in synergistic inhibition on tumor growth in RAS-driven lung cancer [125]. We await future clinical trials to understand the efficacy of this combination in terms of therapeutic limitations. A novel bioactive compound containing a benzothiophene nucleus, DPS2, has been reported to show a potent anti-tumor effect in several colorectal cancer and melanoma cell lines harboring B-Raf or KRAS alterations. DPS2 has shown dual inhibitory action toward Erk and Akt phosphorylation in preclinical models [183]. In cancer cells, DPS2 has been shown to drive apoptosis and also reduce autophagy with high selectivity [183].

A great majority of recent combinatorial strategies targeting MAPK and PI3K/Akt signaling together have been found to be poorly tolerated in clinical studies involving patients with RAS mutations, which diverted the attention to other cellular targets. The inhibition of HSP90, highly expressed in most cancers, in combination with a MEK inhibitor has been identified as a promising therapeutic strategy for KRAS-mutant NSCLC. Several clinical trials have been conducted to examine the effect of AUY922, an HSP90 inhibitor, on solid tumors, and revealed the low cellular toxicity of the agent [184,185]. Owing to the fact that the clinical efficacy of AUY922 remains poor in monotherapies, it has been synergized with trametinib in KRAS-mutant NSCLC and represented a potent anti-tumor activity both in vitro and in vivo [186]. In a later study, the same group has shown that the HSP90 inhibitor, AUY922, suppressed both PI3K/mTOR and Raf/MEK/Erk axes and sensitized cells to PI3K inhibition [187]. In contrast to the combination of MEK and PI3K inhibitors, dual inhibition of HSP90 and PI3K did not induce toxicity in normal cells. Furthermore, AUY922 in combination with the PI3K/mTOR dual inhibitor GSK458 induced apoptosis and reduced compensatory pathway activation in KRAS-mutant NSCLC [187]. Further clinical trials are required to evaluate the efficacy and tolerability of these combinations in patients with RAS mutations. Of note, due to the direct association of HSP90 with specific components of both RAS effector pathways, HSP90-anchored combinations are discussed within the current section.

## 5. Targeting Metabolic Dependencies in Combination with RAS Effectors

One of the key hallmarks of cancer is the metabolic reprogramming of tumor cells, which meet the need of increased biomass by altering their metabolism to grow [188]. RAS signaling promotes metabolic adaptation of tumor cells by coordinating different anabolic processes, including lipid, nucleotide and glycolytic pathways. A growing body of evidence has shown that RAS-driven tumors develop metabolic dependency owing to autophagy to generate metabolic substrates for tumor maintenance [189]. Thus, co-targeting RAS-downstream effectors and the autophagic reliance of the cancerous cells might be an effective strategy to improve tumor response to treatment. Chloroquine and hydroxychloroquine are well-characterized autophagy inhibitors, which function by inhibiting lysosomal acidification. Combinations of autophagy inhibitors with MEK (trametinib and MEK162) [190,191] or Erk (SCH772984) [192] inhibitors or genetic MAPK inhibition [193] have been shown to result in synergistic anti-proliferative effects against multiple refractory RAS-mutant cancer models, including pancreatic, melanoma and lung cancers. These results encouraged the initiation of a number of ongoing clinical trials combining hydroxychloroquine with trametinib (NCT03825289), binimetinib (NCT04132505, NCT04735068) and ulixertinib (NCT04145297) in RAS-addicted cancers, but data about clinical efficacy are not yet available. Although there are some promising results to treat RAS-driven cancers with several combinations, autophagy inhibitors have low potency and require a longer time for efficient treatment. Thus, more potent autophagy inhibitors are currently under development. Given that certain cancer genotypes may be particularly susceptible to autophagy inhibition, challenges and opportunities remain in the selection of patients most likely to benefit from this strategy.

One of the most important metabolisms related to cancer is the mevalonate pathway, which is an essential metabolic pathway for cholesterol biosynthesis. Tumorigenesis requires increased mevalonate pathway flux to meet the need for precursors. The mevalonate synthesis inhibitors, Statins, suppress protein prenylation and mediate several cellular events associated with cancer hallmarks, including proliferation, survival and metastasis. Statins have been previously shown to induce apoptosis by suppressing Erk and Akt activation [194]. Blockade of the mevalonate pathway with the oral administration of statins has been reported as a promising strategy when combined with MEK inhibitors (trametinib and CH5126766) against Akt activation related to MEK inhibitor resistance [195]. On the other hand, it has been shown that inhibition of autophagy sensitizes KRAS-mutant colorectal cancer cells to concurrent use of glycolysis and mevalonate pathway inhibitors [196], suggesting that multiple metabolisms may be targeted simultaneously. The use of kinase inhibitors in alternative drug duos or trios is awaiting evaluation for targeting metabolic dependencies of cancer cells in combination with RAS effectors.

## 6. Summary and Perspectives

Tremendous progress has been made in understanding the genetic architecture, the biological heterogeneity and the distinct molecular pathways driven by RAS oncogenes. Although none of these findings could be successfully extrapolated into cancer therapy yet, future progress will be built on the foundation of this deeper understanding of tumor response. The more detailed assessment of these finely balanced signaling networks could help researchers refine their approach and allow greater focus on rational drug combinations. These observations underscore that a focus should be placed on in-depth optimization of timing, dosing schedules and treatment sequences to remedy toxicities and side effects of drugs. Evidently, there is also still a need to improve clinical tools for accurately stratifying patients based on their molecular status. In this regard, the specific markers associated with drug sensitivity and acquired resistance in aiding therapeutic interventions for RAS-driven cancer entities are of utmost importance.

Beyond small-molecule inhibitors, some innovative therapeutic approaches, such as oncolytic virus-mediated gene-editing [197], mRNA vaccines [198] and novel classes of inhibitors [199], have emerged. RAS-driven tumors have been shown to possess a natural vulnerability to the oncolytic M1 virus, which provides insights into the use of gene-editing oncolytic virotherapy in cancers bearing RAS mutations [200]. Oncolytic viruses are currently under examination and might be a promising alternative for RAS-driven cancer therapy in the upcoming years [201,202]. On the other hand, proteolysis-targeting chimera (PROTAC), comprising ligands of target proteins, E3 ligase-recruiting elements and linkers, has been shown as a potential anticancer therapeutic in KRAS-mutant cancers [203,204]. In addition to these, mRNA vaccines that induce an immune response against specific tumor antigens have been found to be promising in checkpoint-inhibitor-treated melanoma [205]. A phase 1 clinical trial is currently ongoing with a tetravalent RNA-lipoplex cancer vaccine targeting four melanoma-associated antigens in advanced melanoma patients (NCT02410733). There are also improvements in conventional therapies, e.g., stereotactic ablative radiotherapy (SBRT) has been found to be significantly efficient in various solid tumors with remarkable advantages over conventional radiotherapy, and its use in combined modality settings is now considered in various strategies [206].

In the near future, all these efforts will likely bear fruit for the treatment of RAS-driven cancers. Each strategy developed on the basis of scientific research will form another pillar for cancer treatment and serve as an opportunity to develop efficient and well-tolerated combinations in clinical practice.

## Figures and Tables

**Table 1:** Representative inhibitors targeting rat sarcoma virus (RAS) downstream effectors.

Name	Potency	Target	Structure
Dabrafenib/ GSK2118436	IC_50_: 0.6 nM (B-Raf^V600E^) IC_50_: 5 nM (C-Raf)	Raf	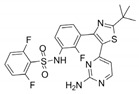
PLX7904	IC_50_: 5 nM (B-Raf^V600E^)	B-Raf^V600E^	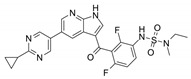
CH5126766/ RO5126766	IC_50_: 8.2 nM (B-Raf^V600E^) IC_50_: 190 nM (B-Raf) IC_50_: 56 nM (C-Raf) IC_50_: 160 nM (MEK)	Raf and MEK1/2	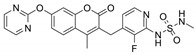
Trametinib/ GSK1120212	IC_50_: 0.92 nM/1.8 nM (MEK1/2)	Mek1/2	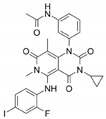
Selumetinib/ AZD6244	IC_50_: 14 nM (MEK1) Kd: 530 nM (MEK2	MEK1/2	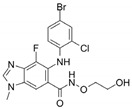
Ulixertinib/ BVD-523	IC_50_: <0.3 nM (Erk2)	Erk1/2	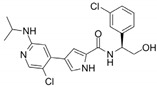
LY3214996	IC_50_: 5 nM (Erk1/2)	Erk1/2	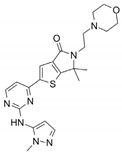
Copanlisib/ BAY80	IC_50_: 0.5 nM (PI3Kα) IC_50_: 0.7 nM (PI3Kδ) IC_50_: 3.7 nM (PI3Kβ) IC_50_: 6.4 nM (PI3Kγ)	Pan-PI3K	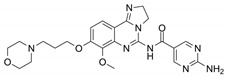
MK-2206	IC_50_: 8 nM (Akt1) IC_50_: 12 nM (Akt2) IC_50_: 65 nM (Akt3)	Akt	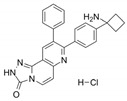
AZD8055	IC_50_: 0.8 nM (mTOR)	mTORC1 and mTORC2	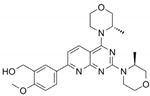

**Table 2:** Active or recruiting clinical trials for the treatment of RAS mutant cancers.

Clinical Trial	Therapy	Phase	Genomic Profile
MEK Inhibitors			
NCT03714958	Trametinib and HDM201 (p53 MDM2 inhibitor)	1	RAS/Raf Mutant and TP53 Wild-type Advanced/Metastatic Colorectal Cancer
NCT03875820	VS-6766 and Defactinib	1	Advanced RAS-mutant Solid Tumors
NCT04303403	Trametinib and Ruxolitinib	1	Advanced RAS-mutant Colorectal Cancer and Pancreatic Adenocarcinoma
NCT03932253	FCN-159	1	Advanced Melanoma Harboring NRAS-aberrant (Ia) and NRAS-mutant (Ib)
NCT01740648	Trametinib Fluorouracil radiation therapy	1	KRAS, B-Raf and NRAS-mutant Rectal Cancers
NCT03681483	VS-6766	1	Advanced KRAS-mutant Lung Adenocarcinomas
NCT03990077	HL-085 and Docetaxel	1	KRAS-mutant NSCLC
NCT03299088	Trametinib and Pembrolizumab	1	Advanced KRAS-mutant NSCLC
NCT02607813	LXH254 and PDR001	1	KRAS-mutant NSCLC, NRAS-mutant Melanoma
NCT02407509	VS-6766 w/o Everolimus	1	Solid Tumors or Multiple Myeloma [115,116]
NCT03704688	Trametinib and Ponatinib	1/2	KRAS-mutant Advanced NSCLC
NCT03170206	Binimetinib and Palbociclib	1/2	Advanced KRAS-mutant NSCLC
NCT02022982	PD-0325901 and Palbociclib	1/2	KRAS-mutant NSCLC, solid tumors
NCT03973151	HL-085	1/2	NRAS-mutant Advanced Melanoma
NCT04409639	Cobimetinib	2	Newly Diagnosed or HMA-treated CMML Patients with RAS Pathway Mutations
NCT01320085	Binimetinib	2	Locally Advanced and Unresectable or Metastatic Malignant Cutaneous Melanoma, Harboring B-Raf^V600^ or NRAS Mutations [120]
NCT04620330	VS-6766 w/o Defactinib	2	Recurrent KRAS-mutant (KRAS-mt) NSCLC
NCT04625270	VS-6766 w/o Defactinib	2	Recurrent Low-Grade Serous Ovarian Cancer (KRAS-mt)
NCT03981614	Binimetinib Palbociclib Trifluridine and Tipiracil Hydrochloride	2	KRAS- and NRAS-mutant Metastatic Colorectal Cancers
NCT01933932	Selumetinib, Docetaxel, Pegylated G-CSF	3	KRAS Mutation-Positive Locally Advanced or Metastatic NSCLC [97,121]
**Erk Inhibitors**			
NCT02857270	LY3214996 w/o Midazolam or Abemaciclib or Nab-paclitaxel or Gemcitabine or Encorafenib or Cetuximab	1	Metastatic Melanoma or NSCLC with B-Raf or RAS Mutations [122,123]
NCT04305249	AZD0364	1	Advanced Solid Tumors and Hematological Malignancies with Alterations in the RAS-MAPK Pathway
NCT02972034	MK-8353 and Pembrolizumab	1	Advanced Malignancies
NCT03698994	Ulixertinib	2	Tumors Harboring Activating MAPK Pathway Mutations
**Vertical Strategies**			
NCT04835805	Belvarafenib, Cobimetinib and Atezolizumab	1	NRAS-mutant Advanced Melanoma Who Have Received Anti-PD-1/PD-L1 Therapy
NCT03284502	Belvarafenib and Cobimetinib or Cetuximab	1	Locally advanced, or metastatic solid tumors with RAS- or Raf-mutation
NCT02974725	LXH254 and LTT462 or Trametinib or Ribociclib	1	Advanced or Metastatic KRAS- or B-Raf-mutant NSCLC or NRAS-mutant Melanoma
NCT03905148	Lifirafenib, Mirdametinib	1/2	Advanced or Refractory Solid Tumors
NCT04417621	LXH254, LTT462, Trametinib, Ribociclib	2	Previously Treated Unresectable or Metastatic B-Raf^V600^ or NRAS-mutant Melanoma
NCT04059224	Trametinib, Dabrafenib	2	Advanced pretreated BRAF^V600^ wild-type/NRAS-mutant melanoma and advanced pretreated BRAF V600 wild-type/NRAS wild-type melanoma
**Metabolic Dependencies**			
NCT03825289	Trametinib and Hydroxychloroquine	1	Metastatic Pancreatic Cancer
NCT04145297	Ulixertinib and Hydroxychloroquine	1	Advanced MAPK-mutant Gastrointestinal Adenocarcinomas
NCT04132505	Binimetinib and Hydroxychloroquine	1	KRAS-mutant Metastatic Pancreatic Cancer
NCT04735068	Binimetinib and Hydroxychloroquine pill	2	Advanced KRAS-mutant NSCLC

## Supplementary Materials

The following is available online. Table S1: Inhibitors and their chemical structures along with their potency.
