# Supplementary material for: Recent Developments in Targeting RAS Downstream Effectors for RAS-Driven Cancer Therapy
Source: Molecules. 2021 Dec 14;26(24):7561. doi: 10.3390/molecules26247561 (PMC8703923; doi:10.3390/molecules26247561)
Supplement: Supplementary file 1 [file molecules-26-07561-s001.zip › molecules-1403063-supplementary.pdf]

Table S1. Inhibitors and their chemical structures along with their potency.

Inhibitor	Description	Chemical Structure	Inhibitor	Description	Chemical Structure
Vemurafenib/<br>PLX4032	B-Raf^{V600E} inhibitor which induces cell autophagy.<br>IC_{50}: 31 nM (B-Raf^{V600E})<br>IC_{50}: 48 nM (C-Raf)	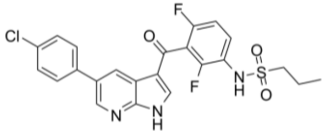	Dabrafenib/<br>GSK2118436	ATP-competitive Raf inhibitor<br>IC_{50}: 0.6 nM (B-Raf^{V600E})<br>IC_{50}: 5 nM (C-Raf)	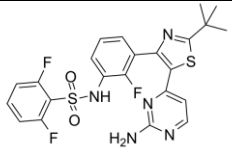
Encorafenib/<br>LGX818	Raf inhibitor with selective anti-proliferative and apoptotic activity in cells expressing B-Raf^{V600E}.<br>IC_{50}: 0.3 nM (B-Raf^{V600E})	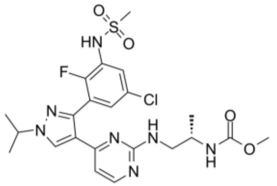	PLX8394	Raf inhibitor<br>IC_{50}: 3.8 nM (B-Raf^{V600E})<br>IC_{50}: 14 nM (wtB-Raf)<br>IC_{50}: 23 nM (C-Raf)	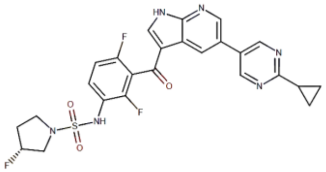
PLX7904	B-Raf inhibitor<br>IC_{50}: 5 nM (B-Raf^{V600E})	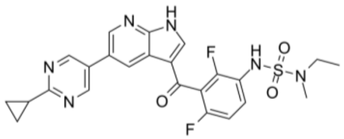	CH5126766/<br>RO5126766	An allosteric dual Raf/MEK inhibitor<br>IC_{50}: 8.2 nM (B-Raf^{V600E})<br>IC_{50}: 190 nM (B-Raf)<br>IC_{50}: 56 nM (C-Raf)<br>IC_{50}: 160 nM (MEK1)	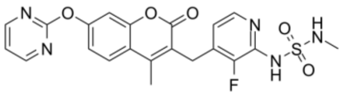
Sorafenib/<br>BAY 43-9006	A multikinase inhibitor of Raf-1 and B-Raf.<br>It inhibits VEGFR-2, VEGFR-3, PDGFR-β, Flt-3 and c-KIT with IC_{50} of 90 nM, 15 nm, 20 nM, 57 nM, 58 nM, respectively.<br>IC_{50}: 6 nM (C-Raf)<br>IC_{50}: 20 nM (B-Raf)	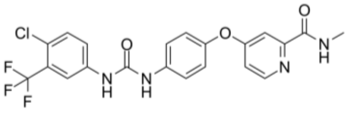	Belvarafenib/<br>HM95573	Pan-Raf kinase inhibitor<br>IC_{50}: 56 nM (wtB-Raf)<br>IC_{50}: 7 nM (B-Raf^{V600E})<br>IC_{50}: 5 nM (C-Raf)	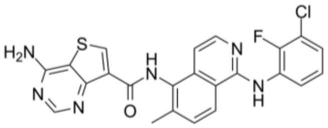
AZ-628	Pan-Raf kinase inhibitor.<br>It inhibits a number of tyrosine protein kinases.<br>IC_{50}: 105 nM (B-Raf)<br>IC_{50}: 34 nM (B-Raf^{V600E})<br>IC_{50}: 29 nM (C-Raf)	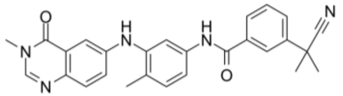	CCT196969	Pan-Raf inhibitor.<br>It inhibits SRC at 26 nM, and LCK at 14 nM.<br>IC_{50}: 100 nM (B-Raf)<br>IC_{50}: 40 nM (B-Raf^{V600E})<br>IC_{50}: 12 nM (C-Raf)	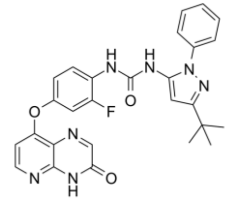

LY3009120/ DP-4978	Pan-Raf inhibitor<br>IC_{50}: 91 nM (B-Raf)<br>IC_{50}: 5.8 nM (B-Raf^{V600E})<br>IC_{50}: 15 nM (C-Raf)	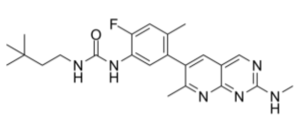	Lifirafenib/ BGB-283	Raf Kinase and EGFR inhibitor<br>IC_{50}: 23 nM (B-Raf^{V600E})<br>IC_{50}: 29 nM (EGFR)	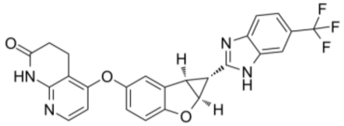
TAK580/ MLN2480	A selective pan-Raf kinase inhibitor.	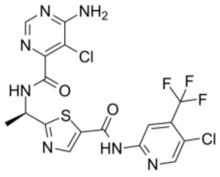	LXH254	A potent B/C-Raf inhibitor.	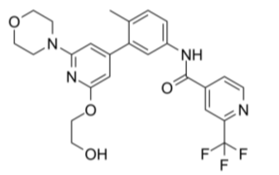
BI-3406	Inhibitor of the interaction between KRAS and Son of Sevenless 1 (SOS1) with IC_{50} of 6 nM.	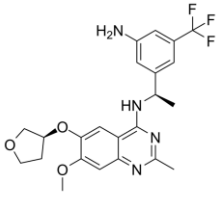	Rineterkib/ LTT462	Raf and ERK1/2 inhibitor.	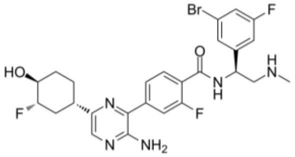
Trametinib/ GSK1120212	MEK1/2 inhibitor<br>It activates autophagy and induces apoptosis.<br>IC_{50}: 0.92/1.8 nM (MEK1/2)	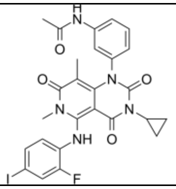	Selumetinib/ AZD6244	MEK inhibitor<br>IC_{50}: 14 nM (MEK1)<br>Kd: 530 nM (MEK2)	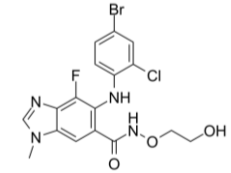
Binimetinib/ MEK162	MEK1/2 inhibitor<br>IC_{50}: 12 nM (MEK1/2)	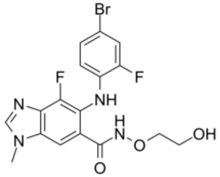	Mirdametinib/ PD0325901	Non-ATP-competitive MEK inhibitor<br>IC_{50}: 0.33 nM	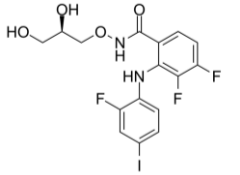
Linsitinib/ OSI-906	Dual inhibitor of the IGF-1 receptor and insulin receptor (IR)<br>IC_{50}: 35 nM (IGF-1)<br>IC_{50}: 75 nM (IR)	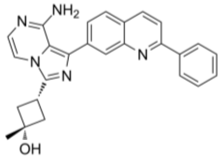	Lapatinib/ GW572016	Inhibitor of the ErbB-2 and EGFR tyrosine kinase domains<br>IC_{50}: 9.8 nM (ErbB-2)<br>IC_{50}: 10.2 nM (EGFR)	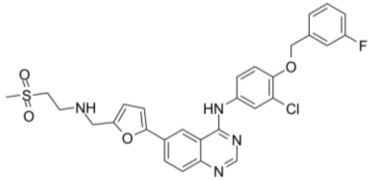

Bemcentinib/<br>BGB324	Axl inhibitor<br>IC_{50}: 14 nM (Axl)	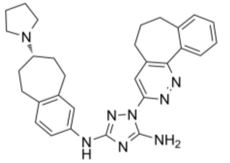	Jnj38877605	An ATP-competitive inhibitor of c-Met<br>IC_{50}: 4 nM (c-Met)	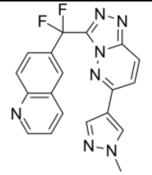
LY3214996	Erk1 and Erk2 inhibitor<br>It potently inhibits cellular p-RSK1 in B-Raf and RAS mutant cancer cell lines.<br>IC_{50}: 5 nM (Erk1/2)	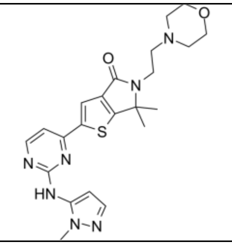	Ravoxertinib/<br>GDC-0994	Erk kinase inhibitor<br>It also inhibits cellular p-RSK1 at 12 nm.<br>IC_{50}: 6.1 nM (Erk1)<br>IC_{50}: 3.1 nM (Erk2)	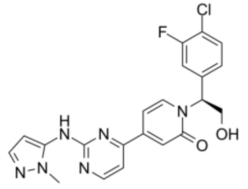
MK-8353/<br>SCH900353	Erk1/2 inhibitor<br>IC_{50}: 23 nM (Erk1)<br>IC_{50}: 8.8 nM (Erk2)	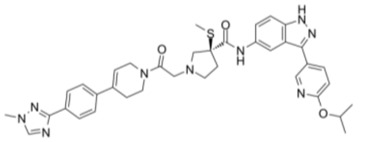	Ulixertinib/<br>BVD-523	ATP-competitive and reversible covalent inhibitor of Erk1/2 kinases,<br>IC_{50}: <0.3 nM (Erk2)	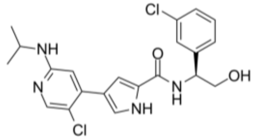
AZD0364/<br>ATG-017	Erk1/2 inhibitor<br>IC_{50}: 0.6 nM (Erk2)	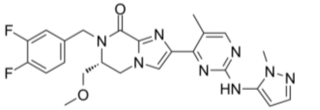	Buparlisib/<br>BKM120	Pan-class I PI3K inhibitor, with IC_{50}s of 52, 166, 116 and 262 nM for p110α, p110β, p110δ and p110γ, respectively.	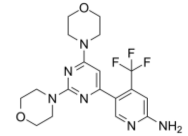
Sonolisib/<br>PX-866	Pan-isoform inhibitor of PI3K<br>IC_{50}: 0.1 nM (p110α)<br>IC_{50}: 1 nM (p120γ)<br>IC_{50}: 2.9 nM (p110δ)	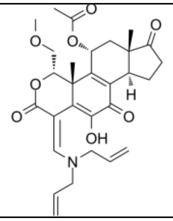	Taselisib/<br>GDC-0032	PI3K inhibitor targets PIK3CA mutations, with Kis of 0.12 nM, 0.29 nM, 0.97 nM, and 9.1 nM for PI3Kδ, PI3Kα, PI3Kγ and PI3Kβ, respectively.	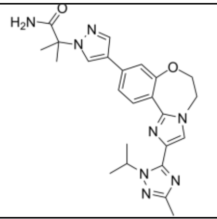
Copanlisib/<br>BAY80	ATP-competitive pan-class I PI3K inhibitor, with IC_{50}s of 0.5 nM, 0.7 nM, 3.7 nM and 6.4 nM for PI3Kα, PI3Kδ, PI3Kβ and PI3Kγ, respectively.	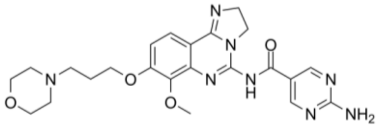	Alpelisib/<br>BYL719	PI3Kα inhibitor (IC_{50}=74 nm)<br>It also inhibits p110α/p110γ/p110δ/p110β with IC_{50}s of 5/250/290/1200 nM, respectively.	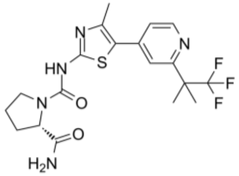

Pictilisib/ GDC-0941	Inhibitor of PI3Kα/δ with an IC50 of 3 nM, with modest selectivity against p110β (11-fold) and p110γ (25-fold)	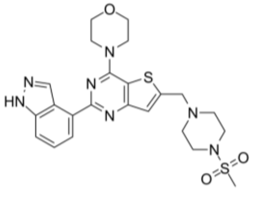	Pilaralisib/ XL147	Class I PI3Ks inhibitor with IC_{50}s of 39 nM, 383 nM, 23 nM and 36 nM for PI3Kα, PI3Kβ, PI3Kγ, and PI3Kδ.	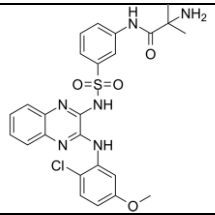
LY294002	PI3K inhibitor with IC_{50}s of 0.5, 0.57, and 0.97 μM for PI3Kα, PI3Kδ and PI3Kβ, respectively. LY294002 also inhibits CK2 with an IC_{50} of 98 nM.	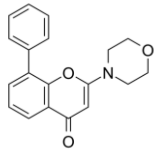	Vistusertib/ AZD2014	ATP competitive mTOR inhibitor with an IC_{50} of 2.81 nM. It inhibits both mTORC1 and mTORC2 complexes.	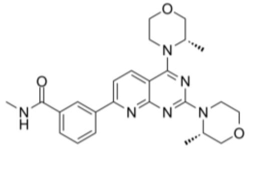
Evorilumus/ RAD001	Rapamycin derivative, mTOR1 inhibitor. Everolimus binds to FKBP-12 to generate an immunosuppressive complex.	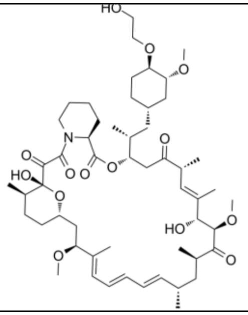	Vorinostat/ SAHA	Pan-inhibitor of HDAC1, HDAC2 and HDAC3 (Class I), HDAC7 (Class II) and HDAC11 (Class IV), with ID50 values of 10 nM and 20 nM for HDAC1 and HDAC3, respectively.	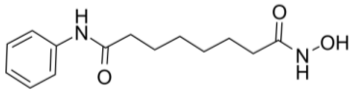
AZD8055	ATP-competitive mTOR kinase inhibitor<br>It inhibits both mTORC1 and mTORC2.<br>IC_{50}: 0.8 nM (mTOR)	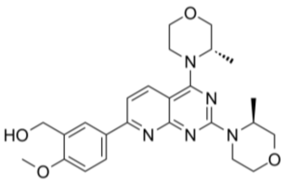	Sapanisertib/ TAK-228	ATP-dependent mTOR1/2 inhibitor<br>IC_{50}: 1 nM (mTOR)	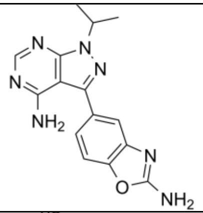
MK-2206	Allosteric Akt inhibitor, with IC_{50}s of 8, 12, and 65 nM for Akt1, Akt2, and Akt3, respectively.	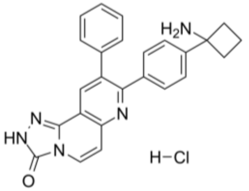	Capivasertib/ AZD5363	Pan-AKT kinase inhibitor respectively.<br>IC_{50}: 3 nM (Akt1)<br>IC_{50}: 7 nMAkt2)<br>IC_{50}: 7 nM (Akt3)	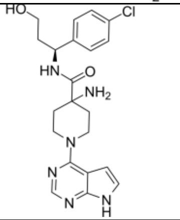

Palbociclib/ PD0332991	CDK4 and CDK6 inhibitor<br>IC_{50}: 11 nM (CDK4)<br>IC_{50}: 16 nM (CDK6)	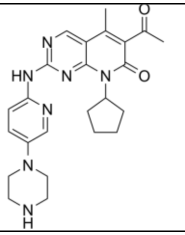	ARS-1620	Atropisomeric selective $KRAS^{G12C}$ inhibitor<br>IC_{50}: 150 nM ($KRAS^{G12C}$)	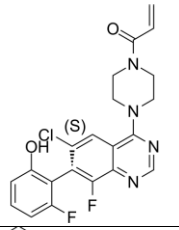
Sotorasib AMG-510	A selective $KRAS^{G12C}$ covalent inhibitor<br>IC_{50}: 1-10 μM ($KRAS^{G12C}$)	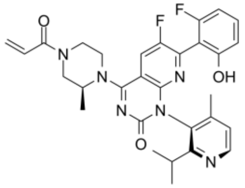	Adagrasib MRTX-849	A covalent inhibitor of $KRAS^{G12C}$<br>IC_{50}: 0.24-1000 nM ($KRAS^{G12C}$)	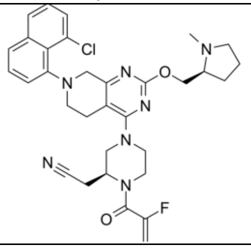
